# Antihistamines Increase Body Mass Index Percentiles and Z-Scores in Hispanic Children

**DOI:** 10.3390/children7120305

**Published:** 2020-12-17

**Authors:** Michelle Saad, Sabeen Syed, Maheen Ilyas, Anatoliy A. Gashev

**Affiliations:** 1 Department of Medical Education, Driscoll Children’s Hospital, Corpus Christi, TX 78411, USA; michelledsaad@gmail.com; 2 Cincinnati Children’s Hospital Medical Center, Department of Gastroenterology, Hepatology and Nutrition, Cincinnati, OH 45229, USA; 3 Department of Gastroenterology, Driscoll Children’s Hospital, Corpus Christi, TX 78411, USA; sabeen.syed@dchstx.org; 4 Department of Chemistry and Biochemistry, City University of New York Hunter College, New York, NY 10065, USA; mayailyas87@gmail.com; 5 Department of Medical Physiology, Texas A&M University Health Science Center, College of Medicine, Bryan, TX 77807, USA

**Keywords:** antihistamine, body mass index, children

## Abstract

The prevalence of childhood obesity has increased over the years in the United States and contributed to a rise in metabolic syndrome and non-alcoholic fatty liver disease (NAFLD). Animal studies suggested the role of histamine blockade on mesenteric lymphatics tone, contributing to weight gain and hepatic steatosis. This study aimed to investigate an association between antihistamines (AH) use in children and obesity. A single-center retrospective cohort study on children with a diagnosis of NAFLD, followed in the gastroenterology clinic, was performed between January 2018 and April 2019. The demographics, medications, and body mass index (BMI) were assessed. Participants were divided into an AH group with documented use and comparison group, antihistamine naïve. Of the 32 participants in the study, 13 used AH, and 19 did not. Antihistamine users had a mean increase in BMI percentile per year of 1.17 compared to a decrease of 0.06 in comparison group (*p* = 0.0008). AH usage correlated with a mean increase in BMI z-score of 0.23 per year, as opposed to a decrease by 0.012 in comparison group (*p* = 0.0016). No difference was found in triglycerides (TG), glucose, and liver enzymes. AH use increases BMI percentiles and z-scores over time and is associated with obesity in children.

## 1. Introduction

The prevalence of obesity has been on the rise since the mid-1980s, now affecting 18.5% of children ages 2–19 years, estimated as 13.7 million per latest Centers for Disease Control and Prevention (CDC) data [1], thereby leading to a 2.7-fold increase in the prevalence of pediatric non-alcoholic fatty liver disease [2] and a higher incidence of metabolic syndrome [3]. In the USA, the Southern states have the highest prevalence of obesity (33.6%) when compared to other regions, particularly in Texas, reaching up to 35% [4] where the Hispanic population, minorities, and low socio-economic groups are most affected [5,6]. Interestingly, areas with uppermost rates of obesity overlap with areas of the highest prevalence of seasonal allergies where antihistamines are prescribed [4].

Nizamutdinova et al. recently elucidated histamine’s vital role as an endothelium-derived relaxing factor on mesenteric lymphatic vessels (MLVs), thereby regulating MLVs’ tone and resistance to lymph flow [7,8]. The blockade of histamine’s intrinsic action modifies the flow-dependent post-prandial adaptation of mesenteric lymphatic contractility and tone. Long-term use of antihistamines leads to increased resistance to lymph flow, accumulation of lipids in the mesentery, and malabsorption [7,8,9]. Moreover, a recent study by Gasheva et al. performed on rats treated with the H1 receptor blocker desloratadine for 16 weeks found an association between H1 blockade and weight gain, increased density of subcutaneous fat, hepatic steatosis, fat malabsorption, and abnormal lipid profiles [9].

The primary objective of this research was to evaluate a potential association between antihistamine use and obesity in children. The secondary goal was to look for a possible association between antihistamine use and laboratory features of metabolic syndrome, i.e., glucose, high-density lipoprotein (HDL), and triglycerides. Children with obesity can be affected by under-evaluated, lymphatic-related side effects of prolonged use of antihistamines.

## 2. Materials and Methods

A retrospective chart review of children who presented to the center’s Pediatric Gastroenterology Department between January 2018 and April 2019 was conducted. The selected participant population had a diagnosis of obesity and pediatric non-alcoholic fatty liver disease (NAFLD), according to the North American Society for Pediatric Gastroenterology Hepatology and Nutrition (NASPGHAN) guidelines, published in 2017 [2]. This participant selection facilitated the assessment of the primary and secondary objectives of the study. Participants included in the study were initially referred from local pediatricians who screened children with overweight or obesity and signs of metabolic syndrome and found elevated alanine aminotransferase (ALT) with pediatric NAFLD suspicion. As clinical practice for NAFLD still varies widely [2], the center excluded alternative etiologies for elevated ALT by further laboratory testing and ultrasound imaging. The procedure of a liver biopsy through interventional radiology was discussed with all participants; however, caregivers refused the biopsy. The participants were managed with intensive lifestyle counseling and close follow-up.

Participants that met the above criteria, with no underlying genetic/metabolic disorders, infections, or malnutrition and persistently elevated ALT as defined by NAFLD’s clinical guidelines, based on sex-specific values, in girls above 22 U/L, and boys above 26 U/L, between the age of 8 to 19 years [2] were included. Participants that did not meet the age range and those with alternate etiologies for elevated ALT, such as viral hepatitis or autoimmune, were excluded. Children with obesity had a BMI ≥ 95th percentile, as per the CDC guidelines [1].

Participants’ BMI numerical values were noted, along with the corresponding BMI percentiles and z-scores for more standardized reporting in growing children. The values were normalized as a change per year due to differences in measurement timing (calculated using the total time of observations). Children with a BMI above the 95th percentile were not further stratified into classes 1, 2, and 3, as the study aimed to track a change over time of the percentiles, in addition to the small sample size limiting further analysis. As pediatric NAFLD is often associated with insulin resistance, obesity, and dyslipidemia, the following variables were assessed in the participant population: age, sex, fasting glucose, triglycerides, HDL, low-density lipoprotein (LDL), and liver enzymes ALT and aspartate aminotransferase (AST) at time of consult.

The medication records documented on Epic Electronic Medical Record (EMR) were examined, especially whether there was current or previous use of antihistamines for every participant. Participants that used antihistamines at any point were placed in the AH group, while those with no identifiable use of antihistamines were in the comparison group. The participants’ following H1 and H2 receptor blockers were included: diphenhydramine, hydroxyzine, cetirizine, loratadine, famotidine, and ranitidine. Other identified medications in some participants’ charts included prescription antipyretics (acetaminophen, ibuprofen) and inhalers (fluticasone, budesonide-formoterol fumarate dihydrate, albuterol sulfate), not known to be associated with weight gain.

The duration of treatment with antihistamines was extracted from the chart. The period of use was noted by looking at the date of addition to the respective participant’s list of medications and subsequent removal later in time. The starting BMI for participants with AH use was the one closest in timing to the beginning of the use of AH noted in the EMR, whereas the final BMI point was the one closest to the reported date of discontinuation of AH on EMR. The starting BMI for participants AH naïve correlated to the time of initial consult visit with the pediatric gastroenterology department, and the final BMI point reflected the last BMI recorded in the growth charts at the time of chart review. The study was conducted in accordance with the Declaration of Helsinki of 1975, revised in 2013. The Ethics Committee of Driscoll Children’s Hospital Institutional Review Board (IRB) approved the project with the identification code 18.034 on the 7th of November 2018. All participant information was appropriately de-identified, compliant with the IRB requirements.

Continuous variables were presented as means with corresponding standard error of the mean (SEM). The variables were compared in participants that used antihistamines to those that did not.

Statistical differences were determined by ANOVA, regression analysis, and Student’s t-test (JMP software version 9.0.2. for Windows, SAS Institute Inc., Cary, NC, USA). A *p*-value < 0.05 with a 95% confidence interval was deemed statistically significant.

## 3. Results

### 3.1. Patient Characteristics

Out of 37 potential subjects identified for the study seen for management of obesity and suspected liver steatosis between January 2018 and April 2019, 32 participants were included in the final analysis that fulfilled criteria for obesity and pediatric NAFLD in the center. The five excluded participants had alternate diagnoses and etiologies for liver enzyme elevation with obesity, including viral hepatitis, autoimmune hepatitis, and systemic lupus erythematosus. Thirteen of the 32 participants (40.62%) received antihistamines, and 19 (59.38%) were antihistamines naïve. Out of 13 total participants that used antihistamines, 10 used H1 receptor blockers (76.9%), and three used H2 receptor blockers (23.1%).

Demographics, including sex, age groups, and clinical characteristics, which met inclusion criteria, are summarized in Table 1. Thirteen participants (40.6%) were boys, and 19 participants (59.4%) were girls. The participants’ self-reported race was extracted from the demographic section of the EMR. Thirty-two participants (100%) were Hispanic. The group of participants that used antihistamines had a median age of 13 years with a mean of 13.4 and a range of 8–18 years. The comparison group had a median age of 16 years, a mean of 14.9, and a range of 10–19 years. 

### 3.2. Clinical and Laboratory Outcomes

Clinical and laboratory variables are highlighted in Table 2. The data collection was complete, with no missing information from EMR. Duration of AH use ranged from 3 months to 64 months, with a median of 12.5 months and a mean of 19.3 months. Starting BMI percentiles and z-scores were compared between the AH and comparison groups, and no statistically significant difference between the two was found. The mean starting BMI percentile for the AH group was 97.62 compared to 98.54 in the comparison group (*p* = 0.099), while the starting mean z-scores for the AH group was 2.16 compared to 2.26 in the comparison group (*p* = 0.36). Participants in the AH group had a mean percentage increase in BMI from baseline per year of 11.42 ± 3.86%, which was higher than the mean percentage increase in BMI from baseline per year in the comparison group 4.57 ± 0.65% (*p* = 0.04). The mean change in BMI percentile in the AH group reflected an increase of 1.17 ± 0.39 percentile per year compared to a decrease of 0.055 ± 0.07 percentile per year in the comparison group (*p* = 0.0008). The AH group’s mean z-score increased by 0.23 ± 0.08 per year compared to a decrease of 0.012 ± 0.01 in the comparison group (*p* = 0.0016).

We found an 8.3-fold increase in BMI percentile/year in AH female users compared to non-AH female users, while in males, the corresponding BMI percentile/year change in AH users was 103.8-fold (*p* = 0.0007, *p* = 0.011, respectively). Changes in BMI z-score/year demonstrated similar trends, with 4.8-fold in females and 89.9-fold in males (data presented in Table 2).

The means of liver enzymes, fasting glucose, triglycerides, and HDL were similar in both groups (*p* = 0.17, *p* = 0.25, *p* = 0.40, *p* = 0.99, *p* = 0.22, respectively). Mean total cholesterol and mean low-density lipoprotein were lower in the group of participants that used antihistamines contrasted to the comparison group with statistical significance (*p* = 0.03, *p* = 0.0058). Ultrasound findings in both groups showed steatosis with varying degrees of hepatomegaly.

## 4. Discussion

Previously, Ratliff et al. performed a study on adults with data based on the 2005–2006 National Health and Nutrition Examination Survey Studies, which included 268 adults taking prescription H1 antihistamines, mainly cetirizine and fexofenadine, along with 599 adults matched by age and gender with controls. Body measurements, glucose, insulin concentrations, and lipid profiles were monitored. Antihistamine users had higher weight, BMI, waist circumference (*p* < 0.001), and insulin levels (*p* < 0.005). They reported an odds ratio of 1.7 of being overweight in both males and females using anti-H1 compared to controls (95% CI, 1.23–2.31), thought to be due to the disruption of insulin and leptin signaling, leading to obesity [10].

In 2019, a study by Stark et al. retrospectively reviewed a large pediatric population prescribed antibiotics, H2 receptor antagonists (H2RA), or proton pump inhibitors (PPI) in the first two years of life and reviewed rates of obesity using a Cox proportional hazards regression model. Antibiotics, H2RA, and PPI use were associated with obesity, especially when participants received a 30-day supply. The authors speculated that acid suppression altered microbiota in early childhood and influenced weight gain [11].

Despite those previous findings in the literature, the link between allergies, intake of antihistamines, and obesity has not been studied clearly and is still a potentially under-recognized pathology. An animal study was recently designed and carried by Gasheva et al. [9] that implemented daily desloratadine treatment in rats for 16 weeks, an equivalent of 8 years in human life [12]. The study results indicate that prolonged intake of desloratadine induced the development of an obesity-like phenotype and signs of metabolic syndrome. More specifically, rats had excessive weight gain with an increase in visceral, subcutaneous fat, and intracapsular brown fat. Moreover, the authors found that animals had high serum triglycerides with signs of deflection towards portal blood, high fasting glucose with signs of insulin resistance, elevated liver to body weight ratio, and liver steatosis suggestive of fatty liver. A dysfunction of mesenteric lymphatic vessels was associated with those findings, precisely high MLV tone, and resistance to flow with lack of proper adaptive reserves to increased lymphatic flow in the post-prandial state [9].

In this retrospective study, an analysis of clinical and laboratory findings from a pediatric population with obesity and non-alcoholic fatty liver disease diagnoses followed by the gastroenterology department was performed. Participants were stratified based on antihistamine use, including any prior or current use, versus non-use. Children who used antihistamines had a BMI percentage increase from baseline per year compared to those that did not use antihistamines.

This study is the first pediatric-focused study looking into antihistamine medication use and obesity beyond the first two years of life. The nature of the study did not allow an assessment of compliance. However, similar to recently performed animal studies, a higher rate of BMI increase over time was found in antihistamine users with obesity, and pediatric NAFLD contrasted to comparison group participants, more prominently so in males. Unlike animal studies, however, triglycerides and fasting glucose were of no statistical significance. Unexpectedly, total cholesterol levels and LDL levels were lower in the study comparison group.

As with similar studies, this study is limited by retrospective design and dependence on documentation and information present on electronic medical records, thereby potentially disregarding non-reported over-the-counter antihistamine use by participants. Another limitation is the lack of data on AH stratification based on their receptor activity (whether anti-H1 or anti-H2) and the comparatively small sample size of the study participants. Moreover, indications for AH use varied but mostly comprised seasonal allergies that can restrict outdoor exercise and activity, thereby independently contributing to weight gain. Another limitation was that the duration of AH use obtained from EMR may not have reflected the participants’ actual adherence to the prescribed therapy.

This study found an association between increased body mass index, its percentiles, and z-scores over time in a pediatric NAFLD population that used antihistamines compared to antihistamine naïve participants. However, the study did not find an association between antihistamine use and laboratory features of metabolic syndrome (glucose, HDL, TG) in this pediatric participant population. Even though papers speculated on various mechanisms over time, the recently elucidated histamine role on mesenteric lymphatics could be one of the critical factors that helps explain the mechanism by which weight gain in antihistamine users happens. With that in mind, the multi-dimensional association between race, age, gender, social-economic status, and the resulting variation in diet, level of physical activity, and use of antihistamines needs to be further elucidated through prospective multivariable studies with a larger number of participants to understand this concept better. Moreover, studies that stratify specific antihistamines used, their duration, and compliance should be performed to help with focused targeted preventive and therapeutic measures.

## 5. Conclusions

Current clinical and animal research data support the notion that millions within the U.S. population are highly likely affected by under-evaluated antihistamines’ side effects, taken to cure various allergic disorders. Therefore, many participants may have developed obesity and metabolic syndrome due to this medication’s prolonged intake.

## Figures and Tables

**Table 1:** Participant characteristics of the study population.

	Total	Antihistamines Use	No Antihistamines Use
Sex, *n* (%)			
Male	13 (40.6)	4 (30.8)	9 (69.2)
Female	19 (59.4)	9 (47.4)	10 (52.6)
Age groups (years), *n* (%)			
8–13	13 (40.6)	7 (53.8)	6 (46.2)
14–21	19 (59.4)	6 (31.6)	13 (68.4)
Race, *n* (%)			
Hispanic	32 (100)	13 (40.6)	19 (59.4)

**Table 2:** Summary of clinical variables stratified by use of antihistamines.

Parameter	Antihistamines Use	No Antihistamines Use	*p* Value
Change in BMI/year (%/year)	11.42 ± 3.86 (*)	4.57 ± 0.65	0.0444
Change in BMI percentile/year (%/year)	1.17 ± 0.39 (*)	−0.05 ± 0.07	0.0008
Change in BMI percentile/year (%/year) in female participants	0.71 ± 0.14 (*)	−0.085 ± 0.13	0.0007
Change in BMI percentile/year (%/year) in male participants	2.18 ± 0.60 (*)	−0.021 ± 0.40	0.011
Change in BMI z-score/year	0.23 ± 0.08 (*)	−0.01 ± 0.01	0.0016
Change in BMI z-score/year in female participants	0.13 ± 0.02 (*)	−0.027 ± 0.02	0.0004
Change in BMI z-score/year in male participants	0.44 ± 0.12 (*)	0.0049 ± 0.08	0.018
Total cholesterol (mg/dL)	147.83 ± 8.11 (*)	170.59 ± 6.18	0.0313
Low-density lipoprotein (mg/dL)	73.75 ± 6.74 (*)	102.35 ± 6.46	0.0058
High-density lipoprotein (mg/dL)	45.33 ± 2.92	40.35 ± 2.65	0.2239
Triglycerides (mg/dL)	147.58 ± 25.93	147.94 ± 15.78	0.9901
Fasting glucose (mg/dL)	97.85 ± 1.96	106.37 ± 8.08	0.3986
Aspartate aminotransferase (U/L)	38.62 ± 6.40	54.42 ± 10.20	0.2494
Alanine aminotransferase (U/L)	65.38 ± 15.53	96.00 ± 15.63	0.1743

Values are means ± SEM; * indicates a significant difference (*p* < 0.05) between study groups (antihistamine users and participants without antihistamine use).

## Abbreviations

AH	Antihistamine
ALT	Alanine Aminotransferase
AST	Aspartate Aminotransferase
BMI	Body Mass Index
CDC	Centers for Disease Control and Prevention
EMR	Electronic Medical Record
H1	Histamine receptor 1
H2	Histamine receptor 2
H2RA	Histamine 2 Receptor Antagonist
HDL	High-Density Lipoprotein
IRB	Institutional Review Board
LDL	Low-Density Lipoprotein
MLV	Mesenteric Lymphatic Vessels
NAFLD	Non-Alcoholic Fatty Liver Disease
PPI	Proton Pump Inhibitor
SEM	Standard Error of the Mean
TG	Triglycerides
